# Diosbulbin B aggravates doxorubicin-induced hepatotoxicity with PI3K/AKT signaling and redox-inflammatory stress responses

**DOI:** 10.3389/fcell.2026.1970882

**Published:** 2026-10-27

**Authors:** Xinran Xie, Yudi Xing, Xiaoyue Liu, Jinghui Zhai, Yanqing Song

**Affiliations:** 1 Department of Pharmacy, Lequn Branch, The First Hospital of Jilin University, Changchun, China; 2 School of Pharmaceutical Sciences, Jilin University, Changchun, China; 3 Department of Clinical Pharmacy, The First Hospital of Jilin University, Changchun, China

**Keywords:** diosbulbin B, doxorubicin, hepatotoxicity, herb–drug interaction, inflammatory stress, mitochondrial dysfunction, oxidative stress-associated cell injury

## Abstract

**Objective:**

This study evaluated whether diosbulbin B (DB) affects doxorubicin (DOX)-induced hepatotoxicity. It further characterized the associated changes in redox-related biochemical indices, mitochondrial membrane potential, inflammatory stress-related proteins, and DNA damage-response-related proteins.

**Methods:**

ICR mice were treated with DOX and/or DB to assess liver injury and serum biochemical changes. LO2 hepatocyte-like cells were used as a simplified *in vitro* model to evaluate cell viability, nuclear morphology, lactate dehydrogenase release, and mitochondrial membrane potential. Bulk RNA sequencing and Western blotting were performed to characterize transcriptomic and protein-expression changes associated with combined DB and DOX exposure.

**Results:**

DB co-administration was associated with lower body weight, comparable hepatic histopathological alterations, and higher serum alanine aminotransferase and aspartate aminotransferase activities in DOX-treated mice. Redox-related biochemical indices showed a non-classical pattern, including increased glutathione levels, decreased malondialdehyde levels, and reduced superoxide dismutase activity in the treatment groups. These changes are indicative of altered redox-related biochemical status rather than direct evidence of a specific oxidative cell-death mechanism. In LO2 cells, DB further reduced cell viability, increased lactate dehydrogenase release, altered nuclear morphology, and enhanced mitochondrial membrane depolarization during DOX exposure. Comparison of the DOX + DB and DOX groups identified 1,221 differentially expressed genes, including 130 upregulated and 1,091 downregulated genes. Enrichment analyses showed terms and pathways related to DNA repair, cell-cycle regulation, chromatin organization, and stress-response processes. Combined treatment was also associated with increased phospho-PI3K/PI3K, phospho-AKT/AKT, p38, TXNIP, IL-6, and IL-8 levels, as well as reduced p53 and GADD45A levels.

**Conclusion:**

DB co-treatment was associated with enhanced DOX-related hepatotoxicity and cytotoxic changes in LO2 cells. Combined exposure was accompanied by mitochondrial membrane depolarization, non-classical redox-related biochemical alterations, increased inflammatory and stress-related protein expression, and reduced p53 and GADD45A abundance. Because direct assays of lipid peroxidation-dependent cell death, ferroptosis, oxytosis, necroptosis, and DNA damage were not performed, the present data do not establish a specific oxidative cell-death mechanism.

## 1 Introduction

Traditional Chinese medicine (TCM) is increasingly used worldwide as a complementary therapeutic approach for chronic diseases and cancer (Li et al., 2023; Zhao et al., 2024). Consequently, the concurrent administration of herbal medicines with conventional drugs has become increasingly common. Although many herbal medicines are generally perceived as safe, some contain intrinsically toxic constituents that may alter the efficacy or toxicity of co-administered drugs through herb–drug interactions (Cheng et al., 2010; Pan et al., 2020; Zhao et al., 2024). Such interactions are of particular concern during chemotherapy because many patients with cancer use herbal medicines during conventional treatment, and potential herb–drug interactions may affect treatment safety (Asiimwe et al., 2021; Cheng et al., 2010). Therefore, systematic evaluation of toxic herbal constituents during chemotherapy is important for improving medication safety.

*Dioscorea bulbifera L.* is a traditional medicinal plant used for the treatment of goiter, inflammation, bleeding, snakebite, and tumors (Guan et al., 2017; Sun et al., 2021; Tan et al., 2022). However, increasing clinical and experimental evidence has demonstrated that D. bulbifera is associated with herb-induced liver injury (Guan et al., 2017; Li et al., 2020; Tan et al., 2022). Diosbulbin B (DB), a major furanoterpenoid constituent of D. bulbifera, is considered one of its principal hepatotoxic components (Yang et al., 2024). DB undergoes cytochrome P450-mediated metabolic activation to form reactive intermediates that can covalently bind to cellular proteins and contribute to hepatocellular injury (Jiang et al., 2017; Yang et al., 2024). This bioactivation-dependent process is regarded as an important mechanism underlying DB-induced hepatotoxicity.

Doxorubicin (DOX) is a widely used anthracycline chemotherapeutic agent whose clinical application is limited by cumulative organ toxicity (Prasanna et al., 2020). In addition to its well-established cardiotoxicity, DOX can induce liver injury through interconnected processes involving reactive oxygen species generation, mitochondrial dysfunction, inflammatory activation, metabolic disturbance, and DNA damage-associated stress responses (Abdel-Latif et al., 2023; AlAsmari et al., 2021; Awadalla et al., 2023). These processes can disrupt redox homeostasis and influence hepatocyte survival under toxic stress. Recent studies have explored pharmacological and biological interventions capable of reducing DOX-associated organ injury (AlAsmari et al., 2021; Mohammed-Rashid and Dyary, 2025).

Most natural-product studies in the context of DOX toxicity have focused on antioxidant, anti-inflammatory, or cytoprotective interventions (Abdel-Latif et al., 2023; AlAsmari et al., 2021; Mohammed-Rashid and Dyary, 2025). In contrast, considerably less is known about toxic herbal constituents that may increase susceptibility to chemotherapy-associated organ injury. A previous study reported that D. bulbifera exposure delayed DOX excretion and aggravated DOX-induced cardiotoxicity and nephrotoxicity (Qu et al., 2019). In addition, a rhizome extract of D. bulbifera was reported to aggravate pirarubicin-associated toxicity by affecting hepatic drug-transporter expression (Sun et al., 2021). However, these studies examined complex plant preparations and focused mainly on cardiotoxicity, nephrotoxicity, or pirarubicin rather than purified DB-associated hepatotoxicity during DOX exposure. Therefore, whether purified DB aggravates DOX-induced hepatotoxicity and alters hepatocellular redox and stress-response networks remains unclear.

Based on this knowledge gap, the present study investigated whether DB aggravates DOX-induced hepatotoxicity using mouse and LO2-cell models. Transcriptomic profiling was used to identify biological processes associated with combined exposure, followed by protein-level evaluation of PI3K/AKT and p38 signaling, inflammatory mediators, and p53/GADD45A-related responses. The study was designed to characterize molecular associations with disrupted hepatocellular stress homeostasis rather than establish a specific form of oxidative cell death.

## 2 Materials and methods

### 2.1 Reagents and chemicals

Diosbulbin B (DB; purity 98%; batch No. c17233278) was purchased from Macklin Biochemical Technology Co., Ltd. (Shanghai, China). Doxorubicin hydrochloride (DOX; purity 98.6%; batch No. 251055) was obtained from MedChemExpress (Monmouth Junction, NJ, United States).

RPMI-1640 medium (Cat. No. F006), fetal bovine serum (FBS; Cat. No. Tx6293), and phosphate-buffered saline (PBS; Cat. No. B015) were obtained from allBio (Shandong, China). Tween-20/TBS solution (Cat. No. 0409A25) was purchased from Beijing Leagene Biotechnology Co., Ltd. (Beijing, China).

Commercial assay kits for alanine aminotransferase (ALT; Cat. No. C009-2-1) and aspartate aminotransferase (AST; Cat. No. C010-2-1) were purchased from Nanjing Jiancheng Bioengineering Institute (Nanjing, China). Assay kits for lactate dehydrogenase (LDH; Cat. No. E-BC-K046-M) and glutathione (GSH; Cat. No. E-BC-K030-M) were obtained from Elabscience Biotechnology Co., Ltd. (Wuhan, China). The malondialdehyde (MDA) assay kit (Cat. No. S0131S) was purchased from Beyotime Biotechnology (Shanghai, China). The superoxide dismutase (SOD) assay kit (Cat. No. A001-3) was purchased from Nanjing Jiancheng Bioengineering Institute.

MTT reagent was obtained from Biofroxx. DAPI staining solution (Cat. No. SL34173550) and rapid blocking buffer (Cat. No. SL35152350) were obtained from Coolaber Science and Technology Co., Ltd. (Beijing, China). The JC-1 mitochondrial membrane potential assay kit (Cat. No. J6004L) was purchased from UElandy Biotechnology Co., Ltd. (Suzhou, China). The bicinchoninic acid (BCA) protein assay kit (Cat. No. WB6501) was obtained from NCM Biotech/Suzhou Xinsaimei Biotechnology Co., Ltd.

Primary antibodies against AKT (Cat. No. YM8463), PI3K (Cat. No. YP0224), phospho-PI3K (Cat. No. YM8045), p38 (Cat. No. YM8267), and NF-κB p65 (Cat. No. YM8209) were obtained from ImmunoWay Biotechnology Company. The phospho-AKT antibody (Cat. No. AA329) was purchased from Beyotime Biotechnology. Antibodies against TXNIP (Cat. No. 18243-1-AP) and UBLCP1 (Cat. No. 12099-1-AP) were purchased from Proteintech Group, Inc. (Wuhan, China). Antibodies against IL-6 (Cat. No. TD6087) and IL-8 (Cat. No. TD6998) were obtained from Abmart Biomedical Inc. (Shanghai, China). Antibodies against GADD45A (Cat. No. 23012166) and GAPDH (Cat. No. 10028231) were obtained from Proteintech Group, Inc. (Wuhan, China), and the p53 antibody (Cat. No. YM8533) was obtained from ImmunoWay Biotechnology Company. The anti-rabbit IgG secondary antibody (Cat. No. A0208) was purchased from Beyotime Biotechnology, and the anti-mouse secondary antibody (Cat. No. CPMC0524010H) was obtained from Starter-bio.com.

Four-percent paraformaldehyde was purchased from Biosharp Life Sciences. Methanol and ethanol were obtained from Sinopharm Chemical Reagent Co., Ltd. (Shanghai, China). All other reagents were of analytical grade.

### 2.2 Animals and treatments

All animal procedures were approved by the Ethics Committee of The First Hospital of Jilin University (Approval No. 2025-694) and were conducted in accordance with institutional guidelines for the care and use of laboratory animals.

Specific pathogen-free male ICR mice, aged 4–6 weeks and weighing 18–22 g, were purchased from Yisi Experimental Animals Co., Ltd. (Changchun, China). Mice were housed under standard laboratory conditions at 22 °C–25 °C and 50%–60% relative humidity under a 12-h light/dark cycle, with free access to standard chow and water. Mice aged 4–6 weeks were selected because this age range is widely used in short-term toxicological studies of hepatotoxic natural products and in mouse models of doxorubicin-associated organ injury. It also corresponds to the young-adult stage commonly used in acute drug-interaction studies. The present protocol was short, with treatments on days 1 and 3 and euthanasia on day 9. The animals were therefore approximately 5–7 weeks of age at the end of the experiment. Preliminary experiments confirmed that this age range tolerated repeated DOX administration while still allowing detectable hepatotoxicity.

After acclimatization, mice were randomly assigned to four groups with eight animals per group: control group (Ctl), doxorubicin-treated group (DOX), doxorubicin plus diosbulbin B-treated group (DOX + DB), and diosbulbin B-treated group (DB). The Ctl group received intraperitoneal normal saline and 0.5% sodium carboxymethyl cellulose by oral gavage on days 1 and 3. The DOX group received DOX at 3 mg/kg by intraperitoneal injection on days 1 and 3. The DOX + DB group received DOX at 3 mg/kg by intraperitoneal injection and DB at 100 mg/kg by oral gavage on days 1 and 3. The DB group received DB at 100 mg/kg by oral gavage on days 1 and 3. The selected doses and administration schedule were based on previous toxicological studies of DB- or Dioscorea bulbifera-associated hepatotoxicity (Tan et al., 2022; Yang et al., 2024) and were optimized in preliminary pilot experiments. DOX was administered at a low, repeated dose of 3 mg/kg on days 1 and 3, giving a cumulative dose of 6 mg/kg. This regimen was chosen to induce measurable hepatotoxicity while limiting systemic mortality. DB was administered at 100 mg/kg, which was well tolerated in preliminary pilot experiments.

Body weight was recorded during the experimental period. On day 9, mice were euthanized, and blood and liver tissues were collected for serum biochemical assays and histopathological examination.

### 2.3 Histopathological examination

Liver tissues were fixed in 4% paraformaldehyde for 24 h, embedded in paraffin, and sectioned into 5-μm-thick slices. Tissue sections were stained with hematoxylin and eosin (H&E) according to standard histological procedures and examined under a light microscope.

Representative images were collected from each group. Histopathological alterations, including inflammatory-cell infiltration and disruption of hepatic architecture, were evaluated.

### 2.4 Serum biochemical and redox-related assays

Blood samples were centrifuged at 3,000 × g for 10 min to obtain serum. Serum ALT, AST, GSH, LDH, and MDA levels, as well as SOD activity, were measured using commercial assay kits according to the manufacturers’ instructions.

The levels of GSH and MDA and the activity of SOD were used as redox-related biochemical indices.

### 2.5 Cell culture

Human LO2 hepatocyte-like cells were cultured in RPMI-1640 medium supplemented with 10% FBS and 1% penicillin-streptomycin. Cells were maintained at 37 °C in a humidified atmosphere containing 5% CO_2_.

Cells in the logarithmic growth phase were used for subsequent experiments. Because LO2 cells possess limited cytochrome P450 metabolic activity, they were used as a simplified *in vitro* model to evaluate the influence of DB on DOX-associated cellular responses rather than to fully reproduce the metabolic activation-dependent hepatotoxicity of DB.

### 2.6 Cell treatment and MTT assay

LO2 cells were seeded into 96-well plates at a density of 5 × 10^3^ cells per well in 100 μL of complete medium and allowed to adhere overnight. To determine the effects of DOX and DB on cell viability, cells were treated with DOX at 1 or 2 μM, DB at 100 or 200 μM, or the corresponding combinations of DOX and DB for 24 or 48 h. For combined treatment, DOX was added 1 h before DB treatment.

After treatment, MTT solution was added to each well, and cells were incubated for 4 h at 37 °C. The medium was then removed, and the resulting formazan crystals were dissolved in dimethyl sulfoxide. Absorbance was measured at 490 nm using a microplate reader. Cell viability was expressed as a percentage of the untreated control.

Based on the MTT results, 2 μM DOX and 200 μM DB were selected for subsequent DAPI staining, JC-1 staining, LDH release assay, and Western blot analysis. For these assays, LO2 cells were divided into four groups: Ctl, DOX, DOX + DB, and DB. Cells in the DOX group were treated with 2 μM DOX, cells in the DOX + DB group were treated with 2 μM DOX and 200 μM DB, and cells in the DB group were treated with 200 μM DB for 24 h. The Ctl group received the corresponding vehicle treatment.

Each experiment was independently repeated at least three times.

### 2.7 DAPI staining

LO2 cells were seeded in 6-well plates and treated with DOX and/or DB for 24 h. Following treatment, cells were washed twice with PBS, fixed with 4% paraformaldehyde for 15 min at room temperature, and stained with DAPI staining solution according to the manufacturer’s instructions.

After washing with PBS, stained cells were observed under a fluorescence microscope. Nuclear morphology was evaluated in representative microscopic fields.

### 2.8 JC-1 staining

Mitochondrial membrane potential was evaluated using a JC-1 mitochondrial membrane potential assay kit according to the manufacturer’s instructions. After treatment with DOX and/or DB for 24 h, LO2 cells were incubated with JC-1 working solution for 30 min at 37 °C and washed with JC-1 buffer.

Fluorescence images were acquired using a fluorescence microscope. Mitochondrial membrane potential changes were evaluated based on the red and green fluorescence signals.

### 2.9 LDH release assay

LO2 cells were treated with DOX, DB, or their combination for 24 h. After treatment, culture supernatants were collected, and LDH activity was measured using a commercial LDH assay kit according to the manufacturer’s instructions.

LDH release was used as an indicator of cell membrane damage.

### 2.10 RNA sequencing and bioinformatic analysis

Total RNA was extracted from LO2 cells in the Ctl, DOX, and DOX + DB groups using TRIzol reagent according to the manufacturer’s instructions. Three biological replicates were included for each group. RNA concentration and purity were determined using a NanoDrop spectrophotometer, and RNA integrity was assessed before library construction.

RNA sequencing libraries were prepared using the U-mRNAseq Library Prep Kit (KAITAI-Bio, Cat. No. AT4221, Hangzhou, China) according to the manufacturer’s protocol. The libraries were sequenced using 2 × 150 bp paired-end sequencing on an Illumina NovaSeq 6000 platform.

Raw sequencing reads were processed using fastp v0.23.4 to remove adapter sequences and low-quality reads. Clean reads were aligned to the human reference genome Homo_sapiens.GRCh38 using HISAT2 v2.2.1. SAMtools v1.16.1 was used to generate BAM files. Gene-level read counts were generated using HTSeq v0.13.5, and gene expression levels were normalized as fragments per kilobase of exon model per million mapped fragments.

Differentially expressed genes were identified using edgeR v3.36.0. Genes with |log_2_ fold change| > 1 and adjusted P < 0.05 were considered significantly differentially expressed.

Venn analysis, principal component analysis, heatmap analysis, and volcano plot analysis were used to visualize the distribution and expression patterns of differentially expressed genes. Gene Ontology and Kyoto Encyclopedia of Genes and Genomes enrichment analyses were performed using clusterProfiler v4.2.0. Gene Set Enrichment Analysis was also performed using clusterProfiler v4.2.0 based on a ranked gene list generated from log_2_ fold-change values. Enriched terms or pathways with adjusted P < 0.05 were considered significant.

### 2.11 Western blot analysis

Total proteins were extracted from cultured LO2 cells using RIPA lysis buffer supplemented with protease and phosphatase inhibitors. Protein concentrations were determined using a bicinchoninic acid protein assay kit. Equal amounts of protein were separated by SDS-PAGE and transferred onto polyvinylidene fluoride membranes.

After blocking with rapid blocking buffer for 15 min at room temperature, membranes were incubated overnight at 4 °C with primary antibodies against AKT, phospho-AKT, PI3K, phospho-PI3K, p38, TXNIP, IL-6, IL-8, GADD45A, p53, UBLCP1, and GAPDH.

After washing with TBST, membranes were incubated with the corresponding horseradish peroxidase-conjugated secondary antibodies for 1 h at room temperature. Protein bands were visualized using enhanced chemiluminescence and quantified using ImageJ software.

The phospho-PI3K level was normalized to total PI3K, and the phospho-AKT level was normalized to total AKT. The levels of p38, TXNIP, IL-6, IL-8, GADD45A, p53, and UBLCP1 were normalized to GAPDH. Relative protein levels were calculated based on integrated optical density values.

### 2.12 Statistical analysis

All statistical analyses were performed using GraphPad Prism version 10.6 (Dotmatics, Boston, MA, United States). Data are presented as the mean ± standard error of the mean (SEM) unless otherwise indicated.

Comparisons between two groups were performed using an unpaired two-tailed Student’s t-test. Comparisons among multiple groups were analyzed using one-way analysis of variance followed by Tukey’s multiple-comparison test or Dunnett’s multiple-comparison test, as appropriate.

A two-sided P value <0.05 was considered statistically significant. Unless otherwise indicated, all cell experiments were independently repeated at least three times. The value of n represents the number of biologically independent animals or independent cell experiments, as specified in the corresponding figure legends.

### 2.13 Immunofluorescence staining

LO2 cells were seeded on glass coverslips and treated with DOX and/or DB for 24 h as described above. After treatment, cells were washed with PBS, fixed with 4% paraformaldehyde for 15 min at room temperature, permeabilized, and blocked. Cells were then incubated overnight at 4 °C with primary antibodies against IL-6, p38, and NF-κB p65, followed by incubation with the corresponding fluorescently labeled secondary antibodies. Nuclei were counterstained with DAPI. Fluorescence images were acquired using a fluorescence microscope, and representative fields from each group were selected for presentation.

## 3 Results

### 3.1 Effects of DOX and DB on body weight, liver histology, and serum biochemical indices in mice

The effects of DOX and DB on body weight and liver-related biochemical indices were first evaluated in mice. The final body weight differed among the four groups. Compared with the control group, the DOX and DOX + DB groups showed lower body weight, whereas the DB group showed a body weight comparable to that of the control group (Figure 1a). During the experimental period, body weight in the control and DB groups remained relatively stable, whereas the DOX and DOX + DB groups showed a downward trend by day 9 (Figure 1b).

**FIGURE 1:**
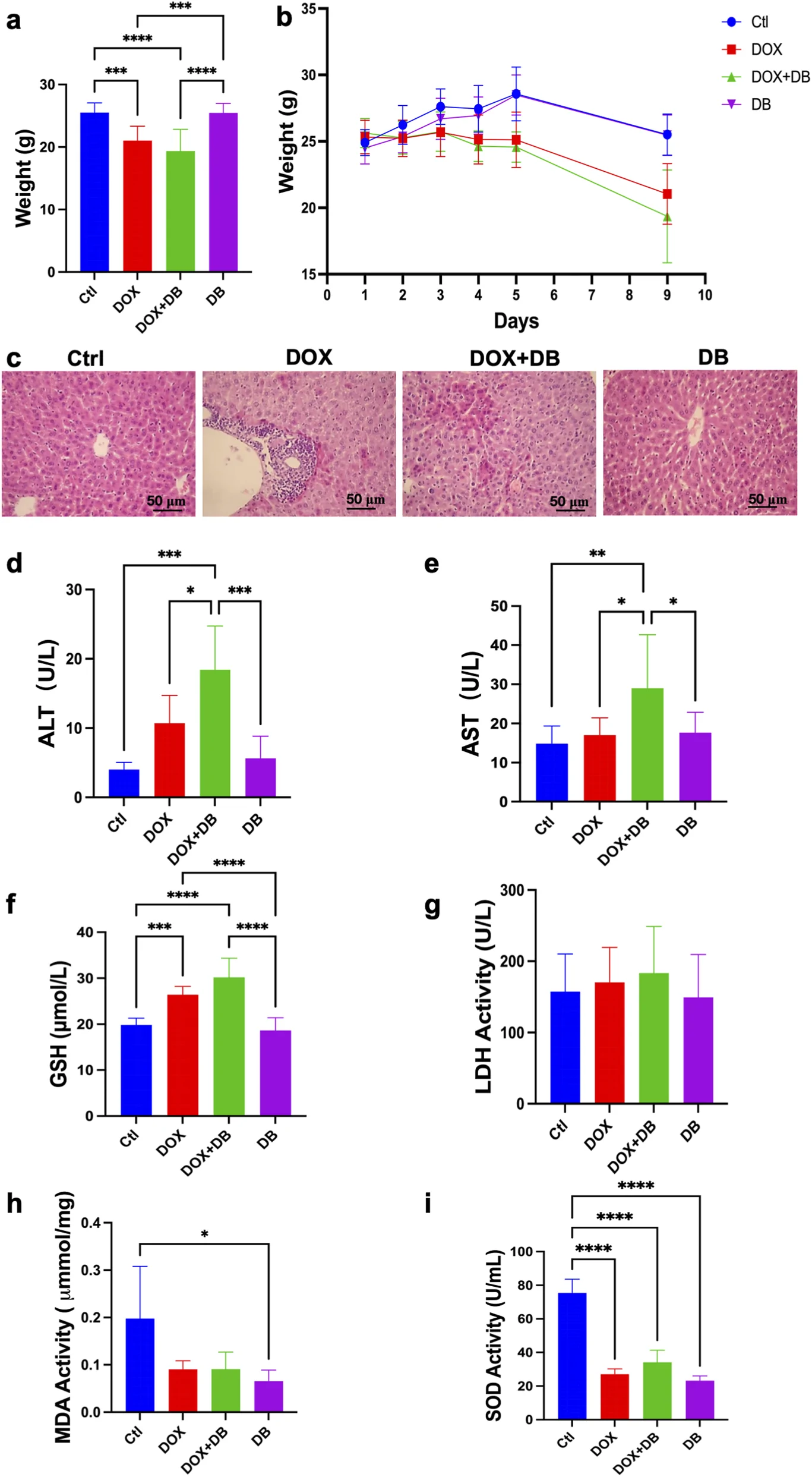
Effects of DOX and DB on body weight, liver histology, and serum biochemical indices in mice. **(a)** Final body weight of mice in the control (Ctl), doxorubicin-treated (DOX), doxorubicin plus diosbulbin B-treated (DOX + DB), and diosbulbin B-treated (DB) groups. **(b)** Body weight changes during the treatment period. **(c)** Representative hematoxylin and eosin **(h,e)**-stained liver sections from each group. Serum levels of **(d)** alanine aminotransferase (ALT), **(e)** aspartate aminotransferase (AST), **(f)** glutathione (GSH), and **(g)** lactate dehydrogenase (LDH). **(h)** Serum malondialdehyde (MDA) levels and **(i)** superoxide dismutase (SOD) activity. Data are presented as the mean ± SEM. *p < 0.05, **p < 0.01, ***p < 0.001, ****p < 0.0001.

Hematoxylin and eosin staining showed relatively preserved liver architecture in the control and DB groups. In the DOX group, inflammatory-cell infiltration and disruption of hepatic architecture were observed. Similar histopathological alterations were also observed in the DOX + DB group (Figure 1c).

Serum alanine aminotransferase (ALT) activity was increased in the DOX group compared with the control group and was higher in the DOX + DB group than in the DOX group (Figure 1d). Serum aspartate aminotransferase (AST) activity also differed among the groups, with the highest level observed in the DOX + DB group (Figure 1e).

Serum glutathione (GSH) levels were higher in the DOX and DOX + DB groups than in the control group, with the highest level observed in the DOX + DB group (Figure 1f). Serum lactate dehydrogenase (LDH) activity showed no obvious difference among the four groups (Figure 1g). Serum malondialdehyde (MDA) levels were lower in the treatment groups than in the control group, with a significant difference marked between the control and DB groups (Figure 1h). Serum superoxide dismutase (SOD) activity was lower in the DOX, DOX + DB, and DB groups than in the control group (Figure 1i).

### 3.2 Effects of DOX and DB on LO2 cell viability, nuclear morphology, mitochondrial membrane potential, and LDH release

The effects of DOX and DB on LO2 cell viability were assessed after 24 and 48 h of treatment. DOX reduced LO2 cell viability compared with the control group. The DOX + DB combination groups showed lower cell viability than the corresponding DOX-treated groups. DB alone caused relatively limited changes in cell viability at the tested concentrations (Figure 2a).

**FIGURE 2:**
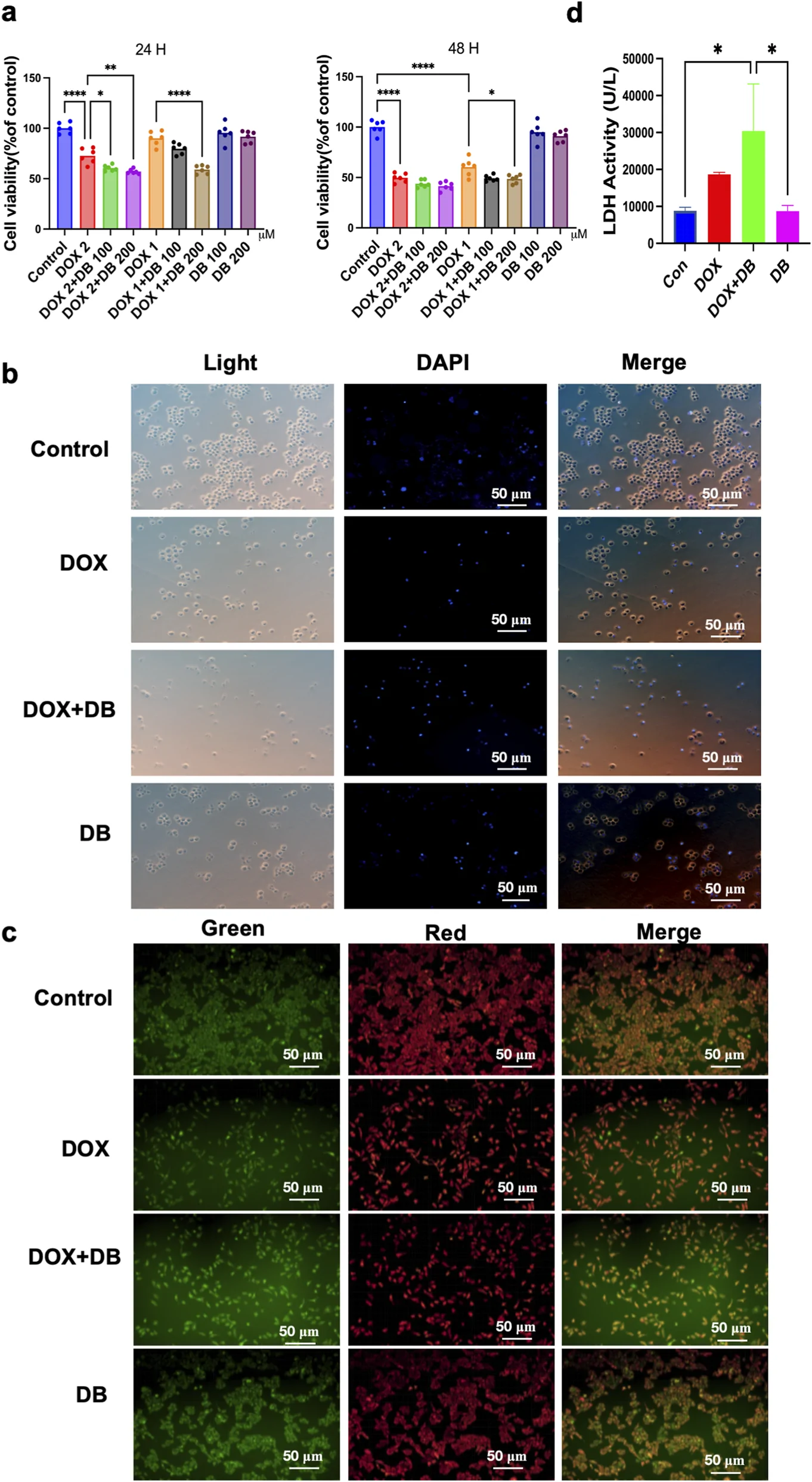
Effects of DOX and DB on LO2 cell viability, nuclear morphology, mitochondrial membrane potential, and LDH release. **(a)** Cell viability of LO2 cells after treatment with DOX and/or DB for 24 and 48 h, as determined by the MTT assay. **(b)** Representative DAPI staining images showing nuclear morphology in each treatment group. **(c)** JC-1 staining showing changes in mitochondrial membrane potential. Red fluorescence represents JC-1 aggregates, whereas green fluorescence represents JC-1 monomers; a decrease in the red/green fluorescence ratio reflects mitochondrial membrane depolarization. **(d)** LDH activity in the culture supernatant. Data are presented as the mean ± SEM. *p < 0.05, **p < 0.01, ***p < 0.001, ****p < 0.0001.

DAPI staining was used to observe nuclear morphology. Compared with the control group, the DOX and DOX + DB groups showed reduced cell density and increased nuclear morphological alterations. Condensed and irregularly shaped nuclei were observed, and the most pronounced changes were found in the DOX + DB group. The DB group showed relatively preserved cell density and nuclear morphology. These features were comparable to those of the control group and clearly distinct from the DOX + DB group (Figure 2b).

JC-1 staining was used to evaluate mitochondrial membrane potential. The control and DB groups showed stronger red fluorescence, whereas the DOX and DOX + DB groups showed increased green fluorescence and reduced red fluorescence. The fluorescence change was more apparent in the DOX + DB group. This finding indicates that combined DOX and DB exposure was associated with more pronounced mitochondrial membrane depolarization than DOX alone. The DB group showed a red/green fluorescence pattern similar to that of the control group (Figure 2c).

LDH activity in the culture supernatant was higher in the DOX group than in the control group and was further increased in the DOX + DB group. The DB group showed LDH activity comparable to that of the control group (Figure 2d).

### 3.3 Transcriptomic profiles of the control, DOX, and DOX + DB groups

RNA sequencing was performed to characterize transcriptomic changes among the control, DOX, and DOX + DB groups. The Venn diagram showed the overlap and unique distribution of differentially expressed genes among the indicated comparisons (Figure 3a). Principal component analysis showed separation among the control, DOX, and DOX + DB groups, indicating distinct transcriptomic profiles in the three groups (Figure 3b).

**FIGURE 3:**
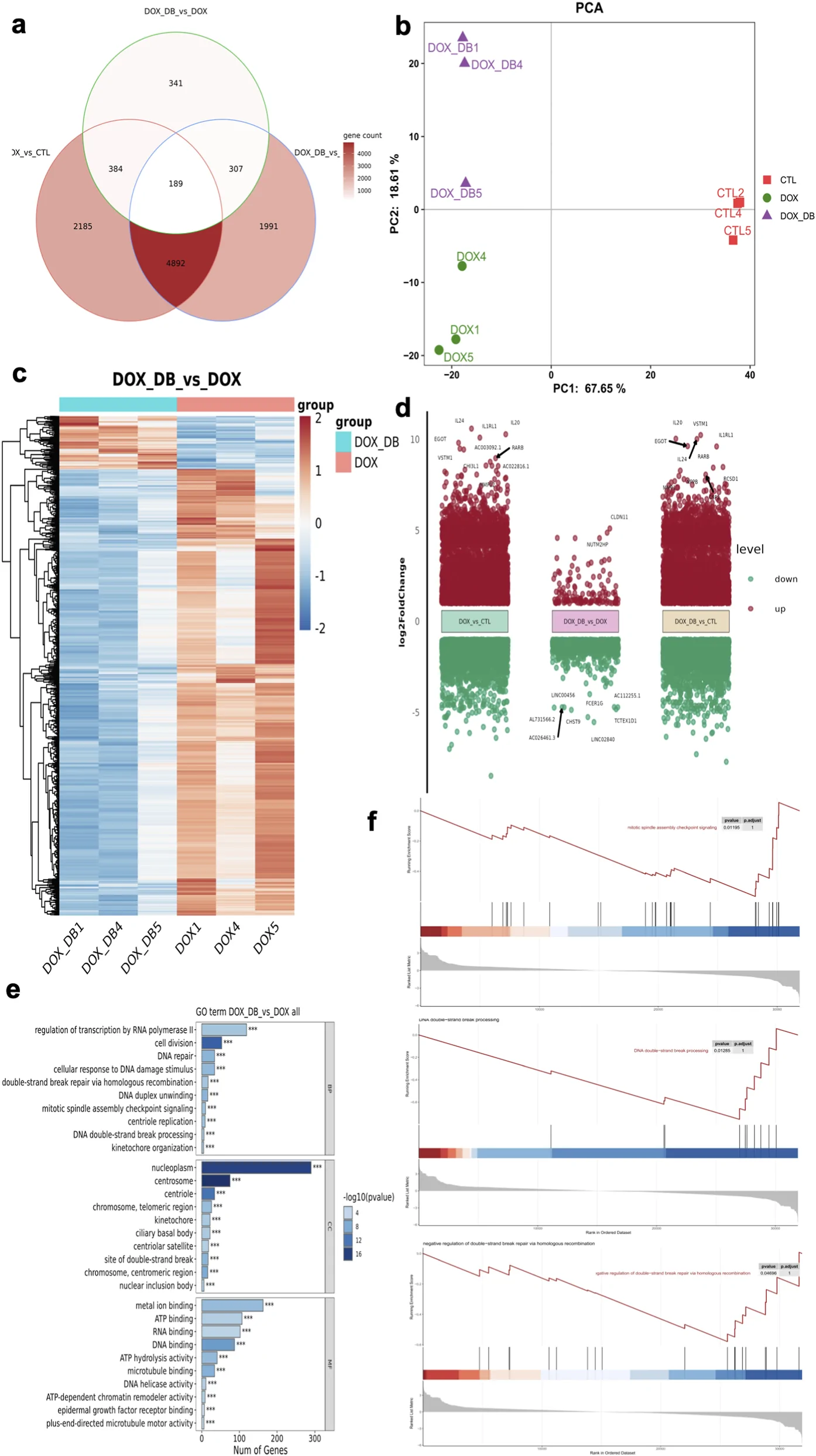
Transcriptomic profiles of LO2 cells after DOX and DB treatment. **(a)** Venn diagram showing shared and unique differentially expressed genes among the indicated comparisons. **(b)** Principal component analysis (PCA) showing the distribution of samples from the Ctl, DOX, and DOX + DB groups. **(c)** Heatmap showing differentially expressed genes between the DOX and DOX + DB groups. **(d)** Overall distribution of upregulated and downregulated genes in the indicated comparisons. **(e)** Gene Ontology (GO) enrichment analysis of differentially expressed genes between the DOX and DOX + DB groups. **(f)** Gene Set Enrichment Analysis (GSEA) plots showing enrichment of gene sets related to mitotic spindle assembly checkpoint signaling, DNA double-strand break processing, and regulation of DNA damage response signal transduction.

The heatmap of differentially expressed genes between the DOX + DB and DOX groups showed distinct expression patterns between the two groups (Figure 3c). The overall distribution of upregulated and downregulated genes in the indicated comparisons is shown in Figure 3d.

In the DOX versus control comparison, 4,462 genes were upregulated and 3,188 genes were downregulated (Supplementary Figure S1a). In the DOX + DB versus DOX comparison, 130 genes were upregulated and 1,091 genes were downregulated (Supplementary Figure S1b).

Gene Ontology enrichment analysis of differentially expressed genes between the DOX + DB and DOX groups showed enriched terms related to transcriptional regulation, cell division, DNA repair, cellular response to DNA damage stimulus, double-strand break repair, homologous recombination, chromatin organization, and mitotic spindle checkpoint signaling (Figure 3e).

Kyoto Encyclopedia of Genes and Genomes pathway enrichment analysis showed enriched pathways including chromosome and associated proteins, membrane trafficking, transcription factors, ubiquitin system, DNA repair and recombination proteins, DNA replication proteins, cellular senescence, NOD-like receptor signaling pathway, cell cycle, platinum drug resistance, nucleocytoplasmic transport, Fanconi anemia pathway, homologous recombination, mitophagy, mineral absorption, and non-homologous end joining (Supplementary Figure S1c).

Gene Set Enrichment Analysis plots showed enrichment profiles for gene sets related to mitotic spindle assembly checkpoint signaling, DNA double-strand break processing, and regulation of DNA damage response signal transduction (Figure 3f).

### 3.4 Effects of DOX and DB on selected signaling and stress-response proteins in LO2 cells

Western blot analysis was performed to examine AKT, p-AKT, PI3K, p-PI3K, p38, TXNIP, IL-6, IL-8, GADD45A, p53, UBLCP1, and GAPDH protein expression in LO2 cells after DOX and/or DB treatment (Figures 4a–h).

**FIGURE 4:**
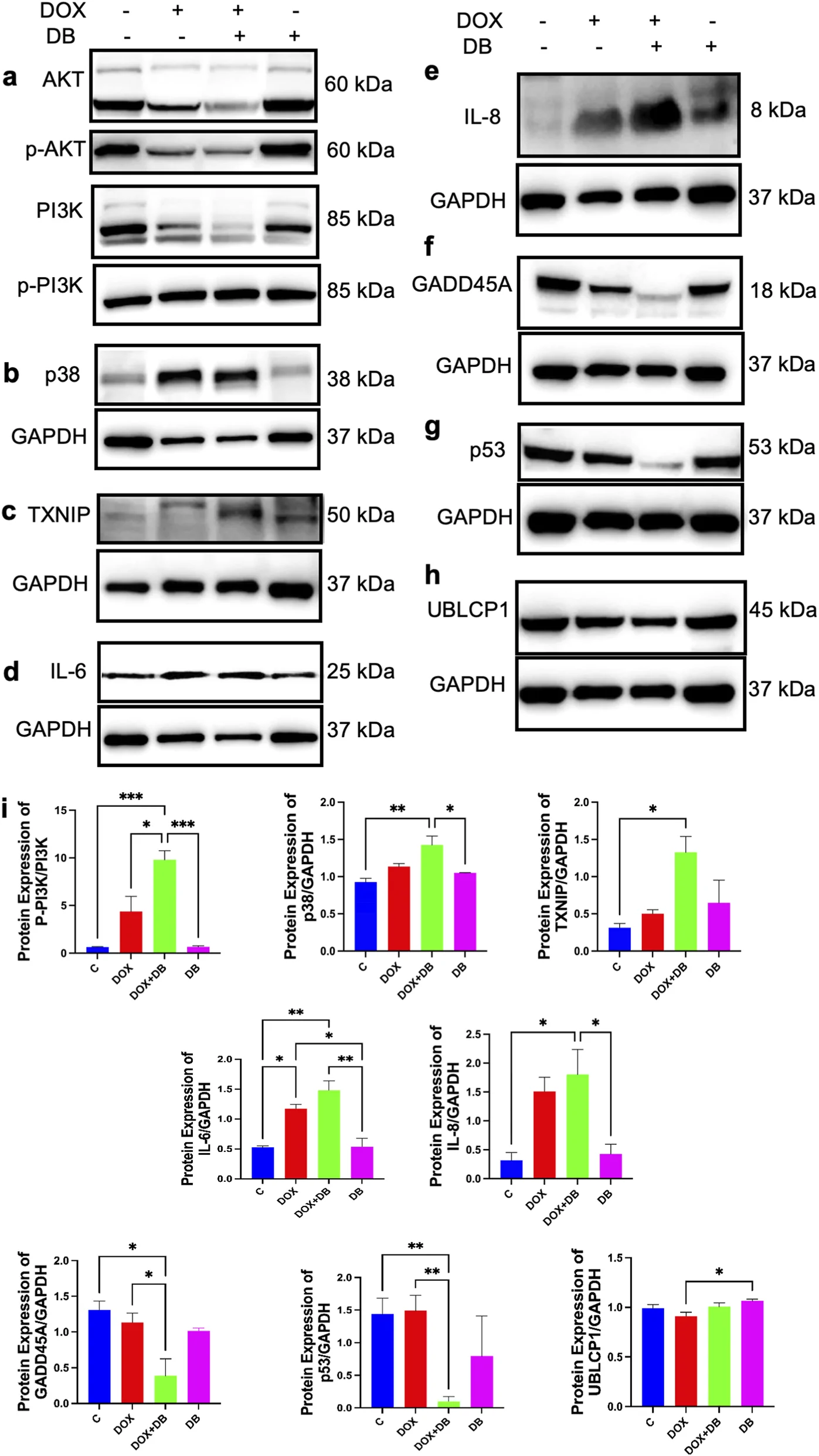
Effects of DOX and DB on selected signaling and stress-response proteins in LO2 cells. **(a–h)** Representative Western blot bands showing the levels of AKT, phosphorylated AKT (p-AKT), PI3K, phosphorylated PI3K (p-PI3K), p38, TXNIP, IL-6, IL-8, GADD45A, p53, and UBLCP1 in LO2 cells treated with DOX and/or DB. GAPDH was used as the loading control. **(i)** Quantitative analysis of the indicated protein levels and phosphorylation-related protein ratio based on integrated optical density. The results show treatment-associated changes in PI3K/AKT-related proteins, p38, TXNIP, IL-6, IL-8, GADD45A, p53, and UBLCP1. Data are presented as the mean ± SEM. *p < 0.05, **p < 0.01, ***p < 0.001, ****p < 0.0001.

The p-PI3K/PI3K ratio was higher in the DOX and DOX + DB groups than in the control and DB groups, with the highest level observed in the DOX + DB group (Figures 4a,i). The p-AKT/AKT ratio was also increased in the DOX and DOX + DB groups, with the highest level observed in the DOX + DB group (Figure 4a).

P38 protein expression was higher in the DOX + DB group than in the other groups, as shown by the p38/GAPDH quantification (Figures 4b,i). TXNIP expression was increased in the DOX and DOX + DB groups compared with the control and DB groups, with a higher level observed in the DOX + DB group (Figures 4c,i).

IL-6 expression was increased in the DOX and DOX + DB groups compared with the control and DB groups (Figures 4d,i). IL-8 Western blot bands were stronger in the DOX and DOX + DB groups than in the control and DB groups (Figure 4e).

GADD45A expression was lower in the DOX + DB group than in the control, DOX, and DB groups (Figures 4f,i). P53 expression was also reduced in the DOX + DB group compared with the control and DOX groups (Figures 4g,i). UBLCP1 expression showed relatively small differences among the four groups, with a significant difference marked between the DOX and DB groups (Figures 4h,i).

Overall, the Western blot results showed treatment-associated changes in PI3K/AKT-related proteins, p38, TXNIP, IL-6, IL-8, GADD45A, p53, and UBLCP1 in LO2 cells after DOX and/or DB exposure.

### 3.5 Immunofluorescence staining of IL-6, p38, and NF-κB p65 in LO2 cells

Immunofluorescence staining was used to visualize representative inflammatory and stress-response markers in LO2 cells after DOX and/or DB treatment. The cellular localization and expression changes of these markers were assessed. Compared with the control group, the DOX and DOX + DB groups showed increased IL-6 immunofluorescence. The strongest signal was observed in the DOX + DB group (Figure 5a). P38 immunofluorescence showed a similar pattern, with the strongest increase in the DOX + DB group (Figure 5b). NF-κB p65 immunofluorescence was also increased in the DOX and DOX + DB groups, with the highest intensity in the DOX + DB group (Figure 5c). The IL-6 and p38 findings are consistent with the Western blot results and support the association between combined DB and DOX exposure and enhanced inflammation- and stress-related protein expression.

**FIGURE 5:**
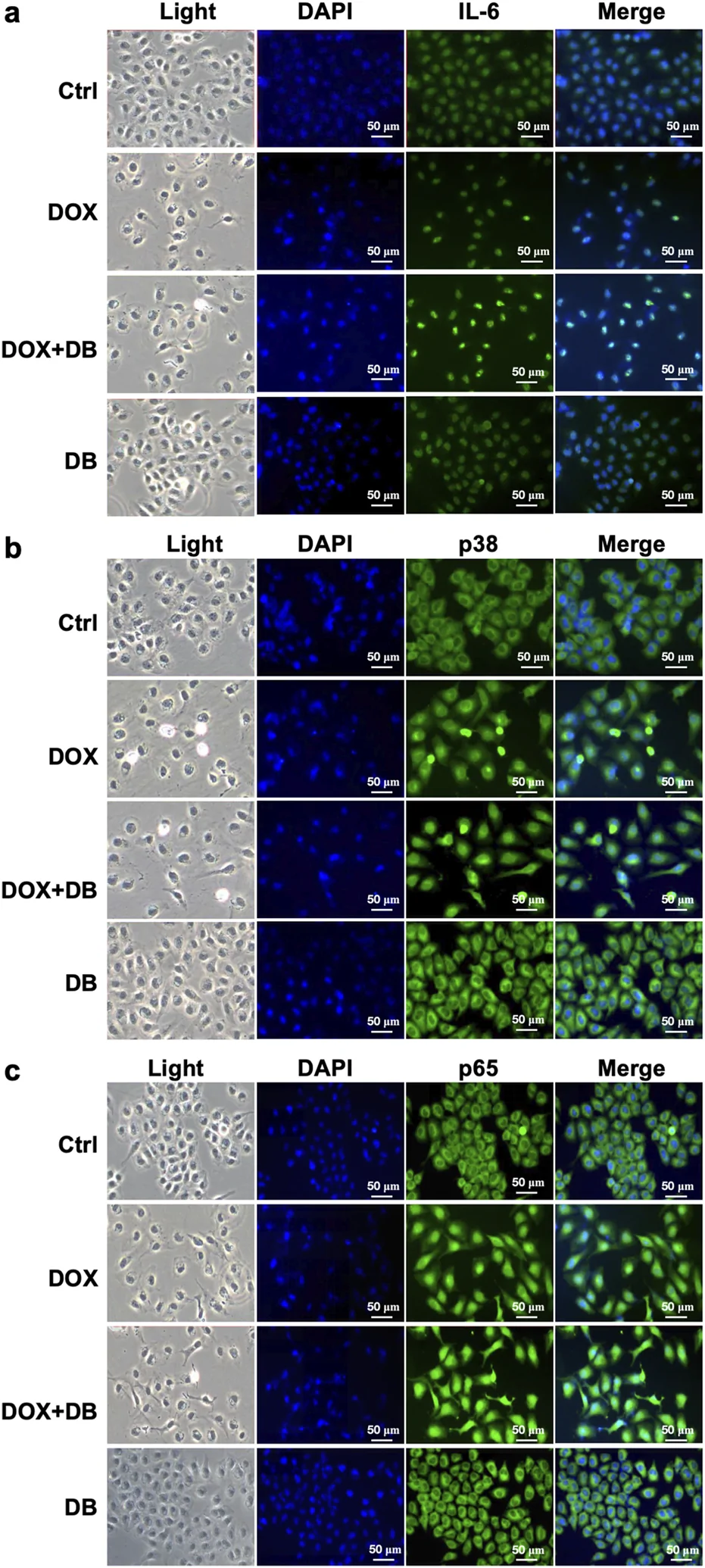
Immunofluorescence staining of IL-6, p38, and NF-κB p65 in LO2 cells. **(a–c)** Representative immunofluorescence images showing the expression and cellular localization of IL-6, p38, and NF-κB p65 in LO2 cells treated with Ctl, DOX, DOX + DB, or DB for 24 h. From left to right: bright-field (Light), DAPI nuclear staining (blue), the indicated marker immunofluorescence (green), and merged images. Compared with the control group, the DOX and DOX + DB groups showed increased immunofluorescence intensity for all three markers, with the most pronounced signal observed in the DOX + DB group.

## 4 Discussion

The present study investigated the effects of combined DB and DOX exposure on liver injury, LO2-cell cytotoxicity, transcriptomic profiles, and selected stress-response proteins. The major findings were as follows: first, DB co-treatment was associated with lower body weight, more evident hepatic histopathological alterations, and higher serum ALT and AST activities in DOX-treated mice. Second, in LO2 cells, combined DOX and DB exposure was associated with reduced cell viability, altered nuclear morphology, mitochondrial membrane depolarization, and increased LDH release. Third, transcriptomic analysis showed distinct gene-expression profiles among the control, DOX, and DOX + DB groups, with a predominance of downregulated genes in the DOX + DB versus DOX comparison. Finally, Western blot analysis showed treatment-associated changes in PI3K/AKT-related proteins, p38, TXNIP, IL-6, IL-8, GADD45A, p53, and UBLCP1.

In the animal experiment, DOX treatment reduced body weight and increased serum alanine aminotransferase and aspartate aminotransferase activities, accompanied by histopathological alterations in liver tissue. These findings are consistent with previous evidence that DOX can induce hepatotoxicity involving oxidative stress, inflammatory responses, mitochondrial dysfunction, apoptosis-associated signaling, and metabolic dysregulation (Abdel-Latif et al., 2023; AlAsmari et al., 2021; Prasanna et al., 2020). The DOX + DB group showed higher serum transaminase activities than the DOX group, together with comparable histological abnormalities. These observations suggest that DB co-treatment was associated with more severe DOX-related liver injury under the present experimental conditions. However, because this study used an experimental mouse model rather than clinical samples, the clinical relevance of this combination should be interpreted cautiously.

DB is a major furanoterpenoid constituent of Dioscorea bulbifera L. and has been recognized as an important toxic component responsible for D. bulbifera-associated liver injury (Li et al., 2020; Tan et al., 2022; Yang et al., 2024). A key feature of DB hepatotoxicity is cytochrome P450-mediated metabolic activation, particularly through CYP3A-associated pathways, which can generate electrophilic reactive metabolites capable of covalent binding to cellular proteins and other nucleophilic biomolecules (Jiang et al., 2017). This bioactivation-dependent toxicity is important for interpreting the present results, especially the cell-based data. Cultured hepatic cell models often show lower cytochrome P450 activity than primary human hepatocytes, which limits their ability to reproduce metabolism-dependent hepatotoxicity (Donato et al., 2013; Rodríguez-Antona et al., 2002). Therefore, LO2 cells in the present study should be regarded as a simplified *in vitro* model for assessing how DB modifies DOX-associated cellular stress and cytotoxicity, rather than as a complete model of DB metabolic activation-dependent hepatotoxicity.

The redox-related biochemical results showed a non-classical pattern. Although the DOX + DB group showed more severe liver injury than the DOX group, serum glutathione levels increased, malondialdehyde levels decreased or remained low, and superoxide dismutase activity decreased across treatment groups. These changes do not support a simple interpretation that combined DB and DOX exposure aggravated liver injury solely through enhanced lipid peroxidation. Redox homeostasis is a dynamic and compartmentalized process, and oxidative stress should not be inferred from a single biochemical marker alone. Instead, oxidative stress has been increasingly understood as a disruption of redox signaling and control, with or without overt molecular damage (Jones, 2006; Sies, 2020). Therefore, the present findings are more appropriately described as redox-related biochemical alterations rather than direct evidence of lipid peroxidation-dependent oxidative cell death.

The increased serum glutathione level observed in the DOX and DOX + DB groups may reflect an adaptive or compensatory response to toxic stress. Glutathione participates in detoxification, thiol redox buffering, and cellular defense against electrophilic and oxidative challenges (Jones, 2006; Lu, 2013). However, serum glutathione does not necessarily reflect the same redox state as intracellular or liver-tissue glutathione pools, and different thiol/disulfide systems may respond differently to toxicants and physiological stimuli. Similarly, the reduced or unchanged malondialdehyde levels may be related to the sampling time point, altered lipid metabolism, tissue-serum differences, or the consumption and turnover of lipid peroxidation products. Thus, the GSH, MDA, and SOD data should be interpreted as evidence of altered redox-related biochemical status rather than as definitive proof of increased or decreased oxidative injury (Sies, 2020). Taken together, the non-classical pattern of increased GSH, decreased SOD activity, and decreased or unchanged MDA levels indicates altered redox-related biochemical status. It does not provide direct evidence of increased oxidative damage or a specific oxidative cell-death mechanism.

The cell-based experiments further showed that DB enhanced several DOX-associated cytotoxic changes in LO2 cells. Combined DOX and DB exposure reduced cell viability more strongly than DOX alone, altered nuclear morphology, increased LDH release, and produced more evident JC-1 fluorescence changes. These results are consistent with increased cellular injury, membrane damage, and mitochondrial membrane depolarization under combined exposure. Mitochondrial dysfunction is an important component of DOX-associated cellular injury and is closely linked to redox imbalance, energy metabolism, and cell-fate regulation (AlAsmari et al., 2021; Prasanna et al., 2020). However, JC-1 staining and LDH release alone do not define a specific form of regulated cell death. Therefore, although the present findings are relevant to oxidative stress-associated cell injury, they do not establish ferroptosis, oxytosis, necroptosis, or any other specific oxidative cell-death modality.

Transcriptomic analysis provided additional information on molecular changes associated with combined exposure. Principal component analysis showed separation among the control, DOX, and DOX + DB groups, indicating that DB co-treatment altered the transcriptional profile of DOX-treated cells. In the DOX + DB versus DOX comparison, 1,221 differentially expressed genes were identified, including 130 upregulated and 1,091 downregulated genes. Functional enrichment analyses showed terms and pathways related to transcriptional regulation, cell division, DNA repair, chromatin organization, DNA damage-response-related processes, cellular senescence, cell cycle, homologous recombination, and non-homologous end joining. These results suggest that combined exposure was associated with broad remodeling of gene-expression programs related to cell-cycle regulation and DNA damage-response-associated pathways. Nevertheless, enrichment analysis reflects coordinated gene-expression changes and should not be interpreted as direct evidence of DNA damage or impaired DNA repair.

The Western blot results showed treatment-associated changes in selected signaling and stress-response proteins. PI3K/AKT-related signals were increased after DOX exposure and were higher in the DOX + DB group. The PI3K/AKT pathway can be activated during oxidative stress and is closely related to cell survival, metabolic regulation, and stress adaptation (Barthel and Klotz, 2005). Therefore, increased PI3K/AKT-related protein signals in the present study may reflect an adaptive or compensatory stress response rather than a direct injury-promoting mechanism. This interpretation is important because PI3K/AKT signaling may have context-dependent roles in toxic injury, survival signaling, and stress recovery. Since pathway inhibitors or genetic interventions were not performed, the present data do not establish whether PI3K/AKT activation contributes causally to injury progression or represents a secondary adaptive response.

P38 protein expression was also increased in the DOX + DB group. P38 MAPK is commonly associated with cellular stress responses, inflammatory signaling, cell-cycle regulation, senescence, and cell-death-related processes (Coulthard et al., 2009). However, the present study measured p38 protein expression rather than p38 phosphorylation. Therefore, the data should be described as increased p38 expression, not as activation of p38 phosphorylation. This distinction is important because phosphorylation status is usually required to infer canonical p38 MAPK activation. Further studies measuring phospho-p38 and using p38 pathway inhibitors would be needed to determine whether p38 signaling functionally contributes to the combined effect of DB and DOX.

TXNIP expression was increased in the DOX + DB group. TXNIP is a stress-responsive protein that interacts with thioredoxin and has been linked to redox regulation, endoplasmic reticulum stress, NLRP3 inflammasome activation, mitochondrial stress, apoptosis, pyroptosis, and inflammatory signaling (Choi and Park, 2023). In the present study, increased TXNIP expression was accompanied by increased IL-6 and IL-8 abundance, suggesting that combined DB and DOX exposure was associated with enhanced stress- and inflammation-related protein expression. However, inflammatory cytokine protein changes alone do not define the upstream regulatory mechanism. Because the present study did not examine NLRP3 inflammasome activation, NF-κB activation, cytokine secretion, or rescue experiments, the TXNIP/IL-6/IL-8 findings should be interpreted as associative protein-expression changes. Accordingly, the present findings are described as changes in inflammation-related protein expression or inflammatory stress-associated responses rather than as definitive activation of a complete inflammatory pathway.

GADD45A and p53 protein levels were reduced in the DOX + DB group. GADD45A is a p53- and BRCA1-regulated stress-response protein involved in cell-cycle checkpoint control, DNA repair, apoptosis, and cellular responses to DNA damage (Zhan, 2005). Thus, reduced GADD45A and p53 abundance is consistent with the transcriptomic enrichment of cell-cycle and DNA damage-response-related pathways. However, the present study did not include direct assays of DNA damage, such as γ-H2AX staining, comet assay, micronucleus assay, or DNA repair functional assays. Therefore, the data support an association between combined DOX and DB exposure and altered p53/GADD45A-related protein expression, but they do not directly demonstrate DNA damage-response impairment. Immunofluorescence validation of p53, GADD45A, TXNIP, and DNA damage markers such as γ-H2AX would further strengthen the study and represents an important direction for future investigations.

UBLCP1 expression showed only limited differences among the treatment groups. UBLCP1 has been reported to function as a phosphatase involved in proteasome-related regulation and cellular protein homeostasis (Guo et al., 2011). In the present study, UBLCP1 was included among the selected proteins examined after transcriptomic screening, but its expression changes were less pronounced than those of TXNIP, IL-6, IL-8, p53, and GADD45A. Therefore, UBLCP1 should be discussed cautiously as a candidate protein whose role in DB- and DOX-associated cellular stress requires further validation.

From the perspective of herb–drug interaction safety, the present findings suggest that DB exposure may modify DOX-associated liver injury and hepatocellular stress responses. Previous studies have mainly focused on natural products or biological interventions that protect against DOX-associated organ injury, including DOX-induced hepatotoxicity or nephrotoxicity (Mohammed-Rashid and Dyary, 2025). In contrast, evidence regarding toxic herbal constituents that aggravate anthracycline-associated toxicity remains limited. A previous study reported that D. bulbifera exposure delayed DOX excretion and aggravated DOX-induced cardiotoxicity and nephrotoxicity by affecting P-glycoprotein expression in mouse liver and kidney (Qu et al., 2019). Another study showed that D. bulbifera rhizome extract aggravated pirarubicin-associated cardiotoxicity by inhibiting hepatic P-glycoprotein and multidrug resistance-associated protein 2 expression (Sun et al., 2021). The present study extends this concern to purified DB and DOX-associated hepatotoxicity, although direct clinical extrapolation remains limited.

The present study is also relevant to the broader field of oxidative stress-associated cell injury, but its scope should be defined carefully. Ferroptosis is generally characterized as an iron-dependent, lipid peroxidation-driven form of regulated cell death that is distinct from apoptosis, necrosis, and autophagy (Dixon et al., 2012; Jiang et al., 2021). Recent expert recommendations emphasize that ferroptosis should be assessed using robust experimental strategies, including appropriate molecular markers, validated modulators, and functional rescue approaches (Mishima et al., 2025). In the present study, lipid ROS, iron accumulation, GPX4, SLC7A11, ACSL4, FSP1, and ferroptosis rescue experiments using ferrostatin-1 or liproxstatin-1 were not examined. Therefore, the current data do not establish ferroptosis or another specific oxidative cell-death mechanism.

Several limitations should be acknowledged. First, the study did not include direct assays of regulated oxidative cell death, including ferroptosis, oxytosis, or ROS-dependent necroptosis. Second, although transcriptomic enrichment involved DNA damage-response-related pathways, direct DNA damage assays were not performed. Third, pathway-intervention experiments using inhibitors, activators, knockdown, or overexpression approaches were not included; therefore, the roles of PI3K/AKT, p38, TXNIP, p53, and GADD45A remain associative. Fourth, LO2 cells cannot fully reproduce DB bioactivation-dependent hepatotoxicity because they are not equivalent to metabolically competent primary hepatocytes. Fifth, pharmacokinetic interactions between DB and DOX were not evaluated, and it remains unclear whether DB alters DOX metabolism, hepatic accumulation, systemic exposure, or excretion. Finally, the selected stress-response proteins were validated in LO2 cells, but corresponding liver-tissue validation was not performed in the current study. In addition, the mice used in the present study were 4–6 weeks old at the start of treatment. Although this age range is commonly used in short-term toxicological studies, validation in older adult mice would further strengthen the generalizability of the findings.

## 5 Conclusion

In conclusion, the present study showed that diosbulbin B co-treatment was associated with enhanced doxorubicin-related liver injury in mice and increased cytotoxic changes in LO2 cells. In mice, combined diosbulbin B and doxorubicin exposure was accompanied by lower body weight, more evident hepatic histopathological alterations, and higher serum alanine aminotransferase and aspartate aminotransferase activities. In LO2 cells, diosbulbin B further reduced cell viability, increased lactate dehydrogenase release, altered nuclear morphology, and enhanced mitochondrial membrane depolarization during doxorubicin exposure.

At the molecular level, combined exposure was associated with broad transcriptomic remodeling, including enrichment of genes related to cell-cycle regulation, chromatin organization, DNA repair, and stress-response-associated processes. Western blot analysis further showed increased PI3K/AKT-related protein signals, elevated p38, TXNIP, IL-6, and IL-8 expression, and reduced p53 and GADD45A abundance. These findings suggest that diosbulbin B may modify doxorubicin-associated hepatocellular stress responses and increase susceptibility to doxorubicin-related hepatic injury.

However, the present study does not establish a specific oxidative cell-death mechanism. Direct assays of ferroptosis, oxytosis, ROS-dependent necroptosis, lipid peroxidation-dependent cell death, DNA damage, pathway causality, and pharmacokinetic interaction were not performed. Further studies using metabolically competent liver models, liver-tissue validation, direct oxidative cell-death assays, DNA damage measurements, pathway-intervention experiments, and pharmacokinetic analyses are needed to clarify the underlying mechanisms and translational relevance of diosbulbin B and doxorubicin co-exposure.

## Data Availability

The data generated in the present study may be requested from the corresponding author.

## Supplementary material

Supplementary Figure S1 Supplementary transcriptomic data. **(a)** Volcano plot showing differentially expressed genes between the DOX and control groups. **(b)** Volcano plot showing differentially expressed genes between the DOX+DB and DOX groups. **(c)** Kyoto Encyclopedia of Genes and Genomes (KEGG) pathway enrichment analysis of differentially expressed genes between the DOX+DB and DOX groups.

## Glossary

ALT: alanine aminotransferase
AST: aspartate aminotransferase
BCA: bicinchoninic acid
CYP: cytochrome P450
DB: diosbulbin B
DEG: differentially expressed gene
DMSO: dimethyl sulfoxide
ECL: enhanced chemiluminescence
DOX: doxorubicin
FBS: fetal bovine serum
GO: Gene Ontology
GSEA: Gene Set Enrichment Analysis
GSH: glutathione
H&E: hematoxylin and eosin
HRP: horseradish peroxidase
IL-6: interleukin-6
IL-8: interleukin-8
JC-1: 5,5′,6,6′-tetrachloro-1,1′,3,3′-tetraethylbenzimidazolylcarbocyanine iodide
KEGG: Kyoto Encyclopedia of Genes and Genomes
LDH: lactate dehydrogenase
MDA: malondialdehyde
MTT: 3-(4,5-dimethylthiazol-2-yl)-2,5-diphenyltetrazolium bromide
NF-κB: nuclear factor-kappa B
PBS: phosphate-buffered saline
PCA: principal component analysis
PVDF: polyvinylidene fluoride
PI3K: phosphoinositide 3-kinase
RIPA: radioimmunoprecipitation assay buffer
SDS-PAGE: sodium dodecyl sulfate-polyacrylamide gel electrophoresis
SEM: standard error of the mean
SOD: superoxide dismutase
TBST: Tris-buffered saline with Tween-20
TCM: traditional Chinese medicine
TXNIP: thioredoxin-interacting protein
UBLCP1: ubiquitin-like domain-containing CTD phosphatase 1.
